# Association of tagSNPs at lncRNA *MALAT-1* with HCC Susceptibility in a Southern Chinese Population

**DOI:** 10.1038/s41598-019-47165-w

**Published:** 2019-07-26

**Authors:** Xiaohui Ji, Junguo Zhang, Li Liu, Ziqiang Lin, Lucheng Pi, Zhifeng Lin, Nana Tian, Xinqi Lin, Sidong Chen, Xinfa Yu, Yanhui Gao

**Affiliations:** 1 Department of Epidemiology and Biostatistics, School of Public Health, Guangdong Pharmaceutical University, Guangzhou, 510310 China; 2 Department of Psychiatry, New York University Langone School of Medicine, One Park Ave, New York, NY 10016 USA; 3 Shunde Hospital of Southern Medical University, Foshan, Guangzhou, China

**Keywords:** Tumour biomarkers, Tumour biomarkers, Genetics research, Cancer epidemiology, Cancer epidemiology

## Abstract

As a long non-coding RNA (lncRNA) and a transcriptional regulator, Metastasis associated lung adenocarcioma transcript-1 (*MALAT-1*) has been reported to be associated with proliferation and metastasis of hepatocellular carcinoma (HCC). However, the effects of *MALAT-1* single nucleotide polymorphisms (SNPs) on HCC remains poorly understood. This study, including 624 HCC cases and 618 controls, aimed to explore the potential associations between three common tagSNPs at *MALAT-1* and HCC risk in a Southern Chinese population. No significant associations were observed between the three tagSNPs and HCC risk under any genetic models after adjusting for potential confounders. Additionally, there were no any significant associations in the stratified analysis, combined effect analysis, and multifactor dimensionality reduction (MDR) analysis. Unification analysis of mediation and interaction on HCC risk further showed that four decomposition of total effects ((controlled direct effect (CDE), the reference interaction effect (INTref), the mediated interaction effect (INTmed), or the pure indirect effect (PIE)) were also not significant. Neither was the association between the *MALAT-1* SNPs and progression factors of HCC, including TNM staging, metastasis, and cancer embolus; Overall, this study suggested that tagSNPs rs11227209, rs619586, and rs3200401 at *MALAT-1* were not significantly associated with HCC susceptibility. Nevertheless, large population-based studies are warranted to further explore the role of *MALAT-1* SNPs in HCC incidence and development.

## Introduction

Hepatocellular carcinoma (HCC) is one of the most common cancers having high morbidity and mortality worldwide^1^, especially in China^2^. An estimated 466,100 new HCC cases occurred in China, with an estimated 422,100 deaths in 2015^2^. The high malignancy and rapid progress of HCC results in most patients to be diagnosed at late stage. Currently, there are no effective treatment strategies, and prognosis remains poor, with a five year survival rate lower than 15%^1,3^. Therefore, it is urgent to explore the pathogenesis of HCC and establish screening mechanism for high risk populations, to help prevent and detect HCC early.

The development of HCC is an extremely complicated process, which results from various environmental and genetic factors^4^. Epidemiological evidences have indicated that chronic infection of hepatitis B virus (HBV) and/or hepatitis C virus (HCV), alcohol drinking, and aflatoxin exposure are major risk factors for HCC^5^. Among genetic factors, non-coding genes, accounting for 98% of the human genome, have been found resulting in many genomic mutations related to the susceptibility of cancers^6^, including HCC^7^. However, it remains not to be fully elucidated the incidence and development of HCC.

Recently, long non-coding RNAs (lncRNAs), a novel class of non-coding RNA, have shown to play significant roles in tumorigenesis, metastasis and prognosis of cancers^8^. Therein, metastasis associated lung adenocarcinoma transcript-1 (*MALAT-1*), a kind of nuclear-retained lncRNA located on 11q36^9^, was upregulated in multiple types of cancers, such as lung cancer^10^, breast cancer (BC)^11^, colon prostate cancer^12^, gastric cancer^13^, and HCC^14,15^. A prior study reported that *MALAT-1* could regulate multiple gene expression through modulating transcription or alternative splicing in multiple cancers^14^. In liver cancer cell lines (HepG2 and Hep3B), *MALAT-1* regulates cancer-related microRNAs (miRNAs), such as mir-574 and mir-20b, contributing to potential targets (such as RAS and MAPK) and Wnt/β-catenin signaling pathway, thus promoting HCC development^16^. Moreover, *MALAT-1* was a proto-oncogene for HCC, which can activate and induce the oncogenic splicing factor SRSF1 through the Wnt pathway^17^. Additionally, lncRNA *MALAT-1* can also serve as a cancer biomarker^18,19^ owing to its ability to facilitate cell proliferation and migration^20^. Although emerging reports have described the function of *MALAT-1* in cancers, knowledge gaps remain regrading the mechanism for cancer pathogenesis.

Single nucleotide polymorphisms (SNPs), which can alter the expression and function of gene, were used to predict disease risks and clinical prognosis^21^. Recently, an increasing number of studies have also explored associations between SNPs at lncRNA *MALAT-1* and cancers risks. For example, the rs3200401 at *MALAT-1* was not associated with BC risk^22^, while the rs619586 variant yielded a lower BC^22^, with no association for lung cancer^23^ and HCC^7^. These results conferred to these SNPs were inconsistent between different cancer types. Additionally, association studies between *MALAT-1* SNPs and HCC were relatively limited. Therefore, we selected three tagSNPs (rs11227209, rs619586 and rs3200401) at *MALAT-1*, and conducted a hospital-based case-control study to systematically explore the association between *MALAT-1* SNPs and development of HCC in the Southern Chinese population.

## Results

### Subjects’ characteristics

Characteristics of 624 HCC cases and 618 controls are presented in Supplementary Table 2. As described previously^24^, the distributions of age and gender were similar in both groups (*P* = 0.429 and 0.752, respectively). HBV infection status was the strongest associated risk factor for HCC (adjusted OR = 15.92, 95% CI = 11.74–21.59). Additionally, alcohol consumption, smoking, ditch water drinking, and family history of cancers were also significantly associated with increased risk of HCC (all *P* < 0.05). Therefore, being regarded as potential confounders, these risk factors were adjusted in the following genetic analyses, as well as age and gender.

### Association between *MALAT-1* SNPs and HCC risk

The genotype distributions of three SNPs in controls were all in agreement with Hardy-Weinberg equilibrium (*P* = 0.671, 0.399, and 0.825 for rs11227209, rs619586, and rs3200401, respectively). Table 1 showed the distributions of genotypes and alleles and their associations with HCC risk. However, no significant associations were observed between all three *MALAT-1* SNPs and HCC susceptibility under any genetic models. Under the dominant model, the adjusted ORs for rs11227209, rs619586, and rs3200401 were 1.29 (95% CI = 0.83–2.03), 1.17 (95% CI = 0.80–1.72) and 0.95 (95% CI = 0.69–1.31), respectively. Combined effect of risk alleles at *MALAT-1* on HCC was also analyzed, however, no significant association was observed (Table 2).

**Table 1:** Associations between *MALAT-1* SNPs and HCC risk in the case-control study.

Genotypes	Cases N = 624 (%)	Controls N = 618 (%)	Crude OR (95% CI)	*P*	Adjusted OR (95% CI)^a^	*P* ^a^
rs11227209						
CC	551 (88.44)	558 (90.44)	1.00		1.00	
CG	68 (10.92)	57 (9.24)	1.21 (0.83–1.75)	0.318	1.26 (0.80–1.99)	0.322
GG	4 (0.64)	2 (0.32)	2.03 (0.37–11.10)	0.416	2.16 (0.30–15.39)	0.442
Dominant	—	—	1.24 (0.86–1.78)	0.254	1.29 (0.83–2.03)	0.260
Recessive	—	—	1.99 (0.36–10.89)	0.429	2.11 (0.30–15.00)	0.457
Additive	—	—	1.24 (0.88–1.75)	0.216	1.28 (0.84–1.93)	0.251
C	1170 (93.90)	1173 (95.06)	1.00		1.00	
G	76 (6.10)	61 (4.94)	1.25 (0.88–1.77)	0.208	1.31 (0.86–2.00)	0.217
rs619586						
AA	522 (83.92)	531 (85.92)	1.00		1.00	
AG	93 (14.95)	82 (13.27)	1.15 (0.84–1.59)	0.382	1.10 (0.74–1.64)	0.627
GG	7 (1.13)	5 (0.81)	1.42 (0.45–4.52)	0.548	2.61 (0.68–10.04)	0.164
Dominant	—	—	1.17 (0.86–1.60)	0.326	1.17 (0.80–1.72)	0.422
Recessive	—	—	1.40 (0.44–4.42)	0.571	2.57 (0.67–9.89)	0.170
Additive	—	—	1.16 (0.87–1.55)	0.301	1.21 (0.85–1.71)	0.288
A	1137 (91.40)	1144 (92.56)	1.00		1.00	
G	107 (8.60)	92 (7.44)	1.17 (0.88–1.56)	0.290	1.22 (0.86–1.75)	0.270
rs3200401						
CC	464 (74.60)	453 (73.42)	1.00		1.00	
CT	149 (23.95)	152 (24.64)	0.96 (0.74–1.24)	0.741	0.97 (0.70–1.35)	0.868
TT	9 (1.45)	12 (1.94)	0.73 (0.31–1.76)	0.485	0.71 (0.24–2.10)	0.537
Dominant	—	—	0.94 (0.73–1.21)	0.636	0.95 (0.69–1.31)	0.765
Recessive	—	—	0.74 (0.31–1.77)	0.499	0.72 (0.24–2.11)	0.544
Additive	—	—	0.93 (0.74–1.17)	0.542	0.95 (0.71–1.26)	0.713
C	1077 (86.58)	1058 (85.74)	1.00		1.00	
T	167 (13.42)	176 (14.26)	0.93 (0.74–1.17)	0.546	0.94 (0.71–1.25)	0.668

Abbreviations: HCC, hepatocellular carcinoma; OR, odds ratio; CI, confidence interval; SNPs, single nucleotide polymorphisms.

^a^Adjusted by age, gender, drinking and smoking status, HBsAg status, history of ditch water drinking, and family history of cancers.

**Table 2:** Combined Effect of the three SNPs in *MALAT-1* on HCC risk.

Number of risk alleles^a^	Cases N = 624 (%)	Controls N = 618 (%)	Crude OR (95% CI)	*P*	Adjusted OR^b^ (95% CI)	*P* ^b^
0–2	522 (84.33)	537 (87.18)	1.00		1.00	
3–4	91 (14.70)	75 (12.18)	1.25 (0.90–1.73)	0.186	1.28 (0.85–1.90)	0.249
5–6	6 (0.97)	4 (0.65)	1.54 (0.43–5.50)	0.504	2.27 (0.50–10.01)	0.285

Abbreviations: OR, odds ratio; CI, confidence interval; SNPs, single nucleotide polymorphisms.

^a^The risk alleles were the G, G, and C for rs11227209, rs619586 and rs3200401 respectively.

^b^Adjusted by age, gender, drinking and smoking status, HBsAg status, history of ditch water drinking, and family history of cancers.

### Stratified analysis of genetic association with HCC risk

We further performed stratified analysis for genetic associations by environmental factors (Supplementary Fig. 1). No significant association between *MALAT-1* rs11227209, rs619586, rs3200401 and HCC susceptibility was found in all subgroups.

### Pair-wise and high-order interactions on HCC risk

No significant interaction was observed between genetic, environmental factors and HCC risk under multiplicative and additive interactions models.

Additionally, high-order interaction analysis were conducted using MDR analysis. However, no evidence of high-order gene-environment interactions on HCC risk were found (Supplementary Table 3 and Fig. 2). The best interaction model was still one-factor model, including HBV.

### Unification analysis of mediation and interaction on HCC risk

Four-way decomposition of total effects (TE) were shown in Table 3. Neither the controlled direct effect (CDE), the reference interaction effect (INTref), the mediated interaction effect (INTmed), nor the pure indirect effect (PIE) was significantly in the association between *MALAT-1* tagSNPs and HCC risk. Additionally, neither significant overall interaction effects between HBV status and tagSNPs, nor significant overall mediated effects role of HBV status was shown. Furthermore, there were no direct or indirect association effects of SNPs on HCC risk. And the total association effects were also not statistically significant.

**Table 3:** Proportions of the effect of SNPs on HCC risk due to mediation and/or interaction with HBV status.

Genotype	Component	Excess Relative Risk (95% CI)	*P*	Proportion Attributable (%)
rs11227209	CDE	−0.030 (−0.117, 0.057)	0.504	11.60
	INTref	−0.219 (−0.760, 0.322)	0.428	85.70
	INTmed	0.002 (−0.046, 0.051)	0.924	−0.92
	PIE	−0.009 (−0.199, 0.180)	0.924	3.61
	**Overall Interaction**	−0.217 (−0.755, 0.322)	0.430	84.78
	**Overall Mediated = Indirect**	−0.007 (−0.148, 0.135)	0.924	2.69
	**Direct**	−0.249 (−0.763, 0.265)	0.343	97.31
	**Total**	−0.256 (−0.78, 0.269)	0.339	100.00
rs619586	CDE	−0.004 (−0.641, 0.633)	0.991	−125.71
	INTref	0.009 (−1.541, 1.559)	0.991	297.53
	INTmed	0.000 (−0.033, 0.032)	0.991	−6.19
	PIE	−0.002 (−0.331, 0.327)	0.990	−65.63
	**Overall Interaction**	0.009 (−1.509, 1.526)	0.991	291.34
	**Overall Mediated = Indirect**	−0.002 (−0.364, 0.359)	0.991	−71.82
	**Direct**	0.005 (−0.908, 0.918)	0.991	171.82
	**Total**	0.003 (−0.548, 0.555)	0.991	100.00
rs3200401	CDE	−0.02 (−0.084, 0.044)	0.539	−6.31
	INTref	0.307 (−0.264, 0.877)	0.292	96.71
	INTmed	0.008 (−0.037, 0.054)	0.723	2.61
	PIE	0.022 (−0.093, 0.137)	0.705	6.99
	**Overall Interaction**	0.315 (−0.273, 0.903)	0.294	99.32
	**Overall Mediated = Indirect**	0.030 (−0.128, 0.189)	0.706	9.60
	**Direct**	0.287 (−0.277, 0.850)	0.319	90.40
	**Total**	0.317(−0.285, 0.919)	0.302	100.00

Abbreviations: CDE: Controlled Direct Effect; INTref: Reference Interaction Effect; INTmed: Mediated Interaction Effect; PIE: Pure Indirect Effect;

Overall Interaction Effect: INTref + INTmed;

Overall Mediated Effect = Indirect Effect: INTmed + PIE; Direct Effect: CDE + INTref;

Total Effect: CDE + INTref + INTmed + PIE = CDE + Overall Interaction + PIE = CDE + INTref + Overall Mediated = Direct + Indirect.

Adjusted by age, gender, drinking and smoking status, HBsAg status, history of ditch water drinking, and family history of cancers.

### Associations between the SNPs and clinicopathological characteristics of HCC

Association analysis between three *MALAT-1* SNPs and TNM stage, metastasis, and cancer embolus were performed (Table 4). Rs11227209, rs619586, and rs3200401 were not significantly associated with metastasis of HCC (adjusted OR = 0.72, 95% CI = 0.35–1.48 for rs11227209, adjusted OR = 0.75, 95% CI = 0.41–1.39 for rs619586, and adjusted OR = 1.08, 95% CI = 0.66–1.77 for rs3200401), cancer embolus of HCC (adjusted OR = 0.60, 95% CI = 0.23–1.58 for rs11227209, adjusted OR = 0.65, 95% CI = 0.28–1.49 for rs619586, adjusted OR = 1.55, 95% CI = 0.87–2.78 for rs3200401), and TNM stage of HCC (adjusted OR = 1.07, 95% CI = 0.55–2.08 for rs11227209, adjusted OR = 0.97, 95% CI = 0.55–1.71 for rs619586 and adjusted OR = 0.96, 95% CI = 0.59–1.56 for rs3200401).

**Table 4:** Associations between the *MALAT-1* SNPs and clinicopathologic characteristics in patients with HCC (N = 624).

Genotypes	TNM stage			Metastasis			Cancer embolus		
	I + II	III + IV	Adjusted OR^a^ (95% CI)	No	Yes	Adjusted OR^a^ (95% CI)	No	Yes	Adjusted OR^a^ (95% CI)
rs11227209									
CC	94 (88.68)	413 (87.69)	1.00	429 (87.73)	93 (90.29)	1.00	461 (88.15)	57 (91.94)	1.00
CG + GG	12 (11.32)	58 (12.31)	1.07 (0.55–2.08)	60 (12.27)	10 (9.71)	0.72 (0.35–1.48)	62 (11.86)	5 (8.07)	0.60 (0.23–1.58)
rs619586									
AA	88 (83.02)	393 (83.62)	1.00	405 (82.99)	89 (86.41)	1.00	436 (83.53)	55 (88.71)	1.00
AG + GG	18 (16.98)	77 (16.38)	0.97 (0.55–1.71)	83 (17.01)	14 (13.59)	0.75 (0.41–1.39)	86 (16.48)	7 (11.29)	0.65 (0.28–1.49)
rs3200401									
CC	75 (72.12)	350 (74.15)	1.00	364 (74.59)	76 (73.79)	1.00	391 (74.90)	42 (67.74)	1.00
CT + TT	29 (27.89)	122 (25.85)	0.96 (0.59–1.56)	124 (25.41)	27 (26.21)	1.08 (0.66–1.77)	131 (25.10)	20 (32.26)	1.55 (0.87–2.78)

Abbreviations: OR, odds ratio; CI, 95% confidence interval; SNPs, single nucleotide polymorphisms.

^a^Adjusted by age, gender, drinking and smoking status, HBsAg status, history of ditch water drinking, and family history of cancers.

## Discussion

Previous studies have demonstrated the roles of lncRNAs as potential oncogenes and critical regulators of tumorigenesis^25,26^. *MALAT-1*, a highly conserved lncRNA in mammals^27^, was proposed to be a potential molecular biomarker of multiple cancers to screen and diagnosis high-risk cancer populations^28^. The highest plasma level of *MALAT-1* was found in HCC patients, followed by other hepatic disease, than in healthy controls, indicating the link of *MALAT-1* over-expression to liver damage^29^. *MALAT-1* expression in HCC tissues was also significantly higher than that in paired non-tumor liver tissues (P < 0.01)^16,30^. Furthermore, *MALAT-1* was upregulated in human liver cancer cell lines compared to normal hepatic cell line (*P* < 0.010), as shown in the gene expression omnibus (GEO) database^16^. The above biological evidences together suggested that *MALTA-1* is an promising susceptibility gene for HCC^31^. However, it is still unknown whether the common variants of *MALTA-1* affect the incidence and development of HCC.

The present study investigated whether tagSNPs of *MALAT-1*, including rs11227209, rs619586, and rs3200401 exhibited effects on the development of HCC. However, no associated evidence between *MALAT-1* tagSNPs and HCC risk were found. Furthermore, there were no statistically significant associations in the combined effects analysis and stratified analysis. The multiplicative and additive interactions and high-order interactions between genes and environments were further explored, but were not significantly associated.

There has been evidence showing that *MALAT-1* rs619586 AG/GG could confer a protective effect on BC in Han Chinese Beijing (CHB) population^22^. However, no associated effect was found for lung cancer in CHS population^23^. Additionally, the association between *MALAT-1* rs619586 and HBV-related HCC risk also did not reach statistical significance in 1300 HBV-positive HCC patients and 1344 HBV persistent carriers (*P* = 0.057)^7^. In this study of CHS population, we further conducted subgroup analysis by HBV status and found that there were still no significant association between rs619586 and HCC risk neither in HBV-positive subgroups nor in HBV-negative subjects. The differences between these association results may be due to dissimilarities in the genetic background of participants and the tissue-specific effect of lncRNA^19^. Peng *et al*. found *MALAT-1* rs3200401 did not significantly associated with BC risk in 487 BC patients and 489 cancer-free controls (*P* = 0.056)^22^. To our knowledge, the effect of rs11227209/rs3200401 on HCC risk was not previously explored. The present study first explored the associations in southern Chinese population, but did not find significant associations between rs11227209/rs3200401 and HCC risk.

Prior studies also reported that *MALAT-1* could promote proliferation, invasion, and metastasis of HCC cells^32,33^, and is an independent prognostic factor for HCC recurrence after liver transplantation^34^. This study further explored the associations between rs11227209, rs619586 and clinic characteristics of HCC, including TNM staging, metastasis, and cancer embolus. The results showed that three tagSNPs were not significantly associated with clinicopathological characteristics of HCC^30^. Nevertheless, additional studies are needed to better understand the mechanism.

The systematic and comprehensive statistical strategy, designing of tagSNP which can capture all common SNPs at *MALAT-1* was adopted in this study. However, limitations still remained. Firstly, potential selection bias may exist because of hospital-based case-control study. Though over 600 cases and 600 controls were included in present study, statistical power was still not enough for subgroup analysis and interaction analysis. Additionally, although many potential epidemiological factors were adjusted for in this study, some unmeasured factors including diabetes should be evaluated in future studies. Moreover, CHS was used as the reference population, which may influence population specificity. Last, it is possible that true HCC-related common SNPs at *MALAT-1* may not be included in the catalog of variants, or *MALAT-1* low frequency variants potentially having stronger effects on HCC risk have not been explored.

In conclusion, this case-control study suggest that three tagSNPs rs11227209, rs619586, and rs3200401 at *MALAT-1* were not associated with HCC risk in this Southern Chinese population. More comprehensive and systematic design (large, population-based, and diverse ethnic population) studies are needed to explore the true *MALAT-1* pathogenic variants on HCC risk.

## Methods

The present study was approved by the Ethics Committee of Guangdong Pharmaceutical University, it was performed in accordance with the approved guidelines of the Declaration of Helsinki. All research and experiments were performed in accordance with relevant guidelines and regulations. Informed consent agreement was obtained from each participant or their legal representatives.

### Study samples

This hospital-based case-control study included 624 HCC patients and 618 cancer-free controls. All participants were consecutively recruited from Shunde hospital of Southern Medical University, in Guangdong, China from September 2010 to October 2014. HCC patients were clinically and histologically diagnosed by pathological examination combined with imaging examination (computed tomography or magnetic resonance imaging). Patients with other cancers were excluded in this study, such as cholangiocarcinoma. Controls were frequency matched with cases based on age (±5 years) and gender, and randomly selected from physical examination populations without history of cancers in the same hospital at the same time when HCC patients were enrolled. Serological markers including HBsAg, anti-HBs, anti-HBc, and anti-HCV were collected from medical records. The information of clinical characteristics of HCC cases, including TNM stage, metastasis status, and cancer embolus, were also reviewed from medical records.

Interviewers were trained to collect epidemiological information, such as gender, age (age at diagnosis for cases), drinking status, smoking status, history of ditch water drinking, and family history of cancer among first-degree relatives. Detail definitions were described previously^24,35^. Additionally, 5 ml whole blood was gathered from each participant following in-person interviews.

### TagSNPs selection and genotyping

To capture all common SNPs across the whole *MALAT-1* gene, we used the design strategy of tagSNPs. There were totally five common SNPs (minor allele frequency (MAF) > 5%) at *MALAT-1*. Among them, rs619586, rs65660 and rs664589 were in the same LD block. Rs11227209 and rs3200401 were the proxies for themselves. Finally, three *MALAT-1* tagSNPs (rs619586, rs11227209 and rs3200401) among the Han Chinese South (CHS) population, were screened out with Haploview version 4.2 software (Cambridge, MA, USA), with the r^2^ > 0.8 and MAF ≥ 5%.

Genomic DNA was extracted from 5 ml whole blood by TIANamp Genomic DNA Kit (Tiangen, Beijing, China) according to the manufacturer. Three tagSNPs were genotyped by TaqMan real-time polymerase chain reaction (PCR) Assay. Primers were designed by AssayDesigner software V3.1. Additional details on PCR primers and probes were previously described^24^. In order to maintain genotyping quality, knowledge of participants’ disease status were blind and 5% random samples were selected to repeat genotyping, with the concordance rate of 100%. The recall rates were 99.84%, 99.84%, and 99.75% for rs11227209, rs619586 and r3200401, respectively (Supplementary Table 1).

### Statistical analyses

Hardy-Weinberg equilibrium (HWE) for genotypes were examined using a goodness-of-fit chi-square test in controls. The differences in frequency distribution of demographic characteristics and genotypes between cases and controls were assessed using student’s t-test (for continuous variables), Person chi-square test or Fisher’s exact probability (for categorical variables). Associations between three SNPs and HCC risk were estimated by odds ratios (ORs) and corresponding 95% confidence intervals (95% CIs) in unconditional logistic regression models. All plausible genetic models, including dominant, recessive, and additive models for these three SNPs, were also assessed. Finally, dominant model was chosen in the following stratified analysis, combined analysis, and interaction analysis. Furthermore, multiplicative and additive gene-environment interactions were estimated using multiplicative term in logistic regression models^36^. We further used unification analysis of mediation and interaction^37,38^ to explore the effect of *MALAT-1* tagSNPs on HCC risk due to mediation and/or interaction with HBV status. Multifactor dimensionality reduction (MDR) analysis was also used to explore potential high-order gene-environment interactions, using MDR V3.0.2 software^39^. Additionally, associations between SNPs and clinicopathological characteristics in HCC patients were also evaluated. A two-tailed *P* < 0.05 was applied as the criterion for statistical significance. Statistical analyses were conducted by software SAS9.4 (SAS Institute, Inc., Cary, NC, USA).

## Supplementary information

Association of tagSNPs at lncRNA MALAT-1 with HCC Susceptibility in a Southern Chinese Population (SREP-18-42781A)_clean version
