# Supplementary material for: Association of tagSNPs at lncRNA MALAT-1 with HCC Susceptibility in a Southern Chinese Population
Source: Sci Rep. 2019 Jul 26;9:10895. doi: 10.1038/s41598-019-47165-w (PMC6659651; doi:10.1038/s41598-019-47165-w)
Supplement: Supplementary file 1 — Association of tagSNPs at lncRNA MALAT-1 with HCC Susceptibility in a Southern Chinese Population (SREP-18-42781A)_clean version [file 41598_2019_47165_MOESM1_ESM.pdf]

Association of tagSNPs at lncRNA *MALAT-1* with HCC Susceptibility in a Southern Chinese Population

Xiaohui Ji[1,*], Junguo Zhang[1,*], Li Liu[1], Ziqiang Lin[2], Lucheng Pi[1], Zhifeng Lin[1], Nana Tian[1], Xinqi Lin[1], Sidong Chen[1], Xinfa Yu[3], Yanhui Gao[1]

[1] Department of Epidemiology and Biostatistics, School of Public Health, Guangdong Pharmaceutical University, Guangzhou 510310, China;

[2] Department of Psychiatry, New York University Langone School of Medicine, One Park Ave, New York, NY 10016;

[3] Shunde Hospital of Southern Medical University, Foshan, Guangzhou, China;

[*] These authors contributed equally to this work;

Correspondence and requests for materials should be addressed to X.Y.(email: yuxfa@126.com) and Y.G. (email: gao_yanhui@163.com).

Supplementary Table 1. The information for MALAT-1 SNPs. SNP, single nucleotide polymorphism; MAF, minor allele frequency; HWE, Hardy-Weinberg equilibrium.

rs ID	Allele	Location in gene	MAF in CHS	MAF in CHB	P for HWE	call rate (%)
rs11227209	C/G	intronic	0.08	0.10	0.671	99.84
rs619586	A/G	intronic	0.11	0.16	0.399	99.84
rs3200401	C/T	intronic	0.16	0.12	0.825	99.75

Supplementary Table 2. Characteristics of HCC cases and controls enrolled in this study. Abbreviations: HCC, hepatocellular carcinoma; NA, not available; SD, standard deviation. [a] The subjects have a family history of cancers in the first-degree relatives. [b] Metastasis status and cancer embolus were determined at first diagnosis of hepatocellular carcinoma. [c] Adjusted by age, gender, drinking and smoking status, HBsAg status, history of ditch water drinking, and family history of cancers.

Characteristics	Cases N = 624 (%)	Controls N= 618 (%)	Crude OR (95% CI)	*P*	Adjusted OR[c] (95% CI)	*P*[c]
Age (year, mean ± SD)	55.42 ± 11.82	55.95 ± 12.08	1.00 (0.99-1.01)	0.429	-	-
Gender						
Female	83 (13.30)	86 (13.92)				
Male	541 (86.07)	532 (86.08)	1.05 (0.72-1.46)	0.752	-	-
Drinking status						
Never	275 (44.07)	341 (55.18)				
Ever	349 (55.93)	277 (44.82)	1.56 (1.25-1.95)	<.001	1.62 (1.184-2.22）	0.003
Smoking status						
Never	200 (32.05)	268 (43.37)				
Ever	424(67.95)	350 (56.63)	1.62 (1.29-2.05)	<.001	1.49 (1.05-2.12）	0.026
HBsAg status						
Negative	163 (26.16)	512 (82.85)				
Positive	460 (73.84)	106 (17.15)	13.63 (10.35-17.95)	<.001	15.92 (11.74-21.59）	<.0001
History of ditch water drinking						
Never	261 (41.83)	336 (54.37)				
Ever	363 (58.17)	282 (45.63)	1.66 (1.32-2.07)	<.001	1.74 (1.30-2.32）	<.0001
Family history of cancers[a]						
No	451 (72.28)	532 (86.08)				
Yes	173 (27.72)	86 (13.92)	2.37 (1.78-3.16)	<.001	2.08 (1.46-2.95）	<.0001
TNM stage						
I+II	106 (16.99)					
III+IV	472 (75.64)					
NA	46 (7.37)					
Metastasis status[b]						
No	490 (78.53)					
Yes	103 (16.51)					
NA	31 (4.96)					
Cancer embolus[b]						
No	524 (83.97)					
Yes	62 (9.94)					
NA	38 (6.09)					

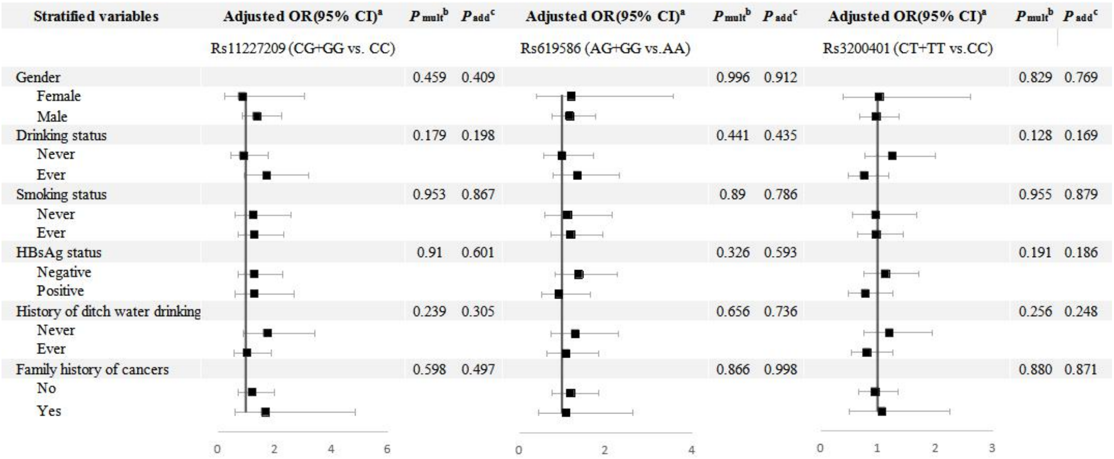

Supplementary Figure 1. Stratified analysis and interaction of *MALAT-1* SNPs with risk factors on HCC. [a] Adjusted by age, gender, drinking and smoking status, HBsAg status, history of ditch water drinking, and family history of cancers. [b] *P* values for interaction between genetic and environmental factors were calculated by multiplicative interaction model. [c] *P* values for interaction between genetic and environmental factors were calculated by additive interaction model.

Supplementary Table 3. Multifactor dimensionality reduction (MDR) analysis for prediction of HCC risk. Abbreviations: CVC, cross-validation consistency. [a] best interaction model; [b] *P* for permutation test.

Interaction Models	Training accuracy	Testing accuracy	CVC	P^b
HBsAg status[a]	0.7836	0.7836	10/10	<0.001
HBsAg status and rs619586	0.7844	0.7836	9/10	<0.001
HBsAg status, age and rs619586	0.7861	0.7836	9/10	<0.001

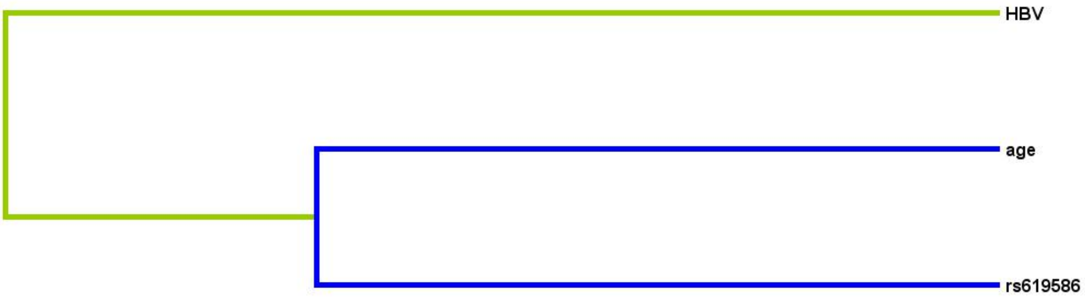

Supplementary Figure 2. Dendogram of three-factor interaction model for predicting HCC risk
